# Dilemmas of adult woman with 46,XY disorders of sexual development: A case report

**DOI:** 10.1016/j.ijscr.2023.109004

**Published:** 2023-10-27

**Authors:** Kevin Yonathan, Irfan Wahyudi, Arry Rodjani, Gerhard Reinaldi Situmorang

**Affiliations:** Department of Urology, Faculty of Medicine, Universitas Indonesia – dr. Cipto Mangunkusumo National General Hospital, Jakarta, Indonesia

**Keywords:** Ambiguous genitalia, Disorders of sexual development, Gender assignment, Infertility, Social stigma

## Abstract

**Introduction and importance:**

Disorders of sexual development (DSD) are a rare but disastrous condition. A plethora of biological, psychological, and ethical problems surround the management of DSD cases.

**Case presentation:**

A case of 25-year-old woman with a complaint of primary amenorrhea was presented. She wanted to be examined as she had planned to be married. However, examinations revealed that she had 46, XY karyotype, complete androgen insensitivity syndrome, and vaginal agenesis. After several family meetings, the patient, her family, and her partner decided that she would remain as a woman despite the results.

**Clinical discussion:**

Disorders of sexual development are not only about the biological complexities, but also the psychological, social, and ethical dilemmas faced by both the patient and clinicians. A coordinated care team plays a crucial role in addressing the unique challenges presented by DSD, particularly for those diagnosed later in life.

**Conclusion:**

Comprehensive and multidisciplinary approach is essential for managing DSD patients.

## Highlights

• Disorders of sexual development (DSD) cases require complex diagnosis and multidisciplinary approach.
• The late diagnosis of DSD is associated with a myriad of biological, psychological, and social problems.
• Surgical and hormonal interventions regiment and timing remain a topic of debate for clinicians.

## 1 Introduction

The development of sexual organs requires perfectly timed interaction between various factors. Should any part of the factors fails to perform properly, the person would be affected with a condition called disorders of sexual development (DSD) [1]. Previously called “intersex disorders”, DSD is a broad term for indicating congenital conditions with atypical development of chromosomal, gonadal, or anatomical sex [2]. Previous data have shown that the prevalence of genital ambiguity is in the range of 1:2000 to 1:4500. Moreover, the prevalence of XY females is 6.4:100,000 live born females and the prevalence of androgen insensitivity is 4.1:100,000 live born females [3]. As of 2023, there are four published consensus statements regarding investigation and management of DSD [2]. However, the investigation and management of DSD vary greatly according to the local infrastructure and social norms [4].

There are various biological, psychological, social, and ethical problems complicating the situation of those affected by DSD [[5], [6], [7]]. It is even more pronounced on those diagnosed at a later part of life and having learned that their examination results showed the characteristics of opposite sex [8]. This study aims to present the dilemmas faced by clinicians and patient of DSD case. This case report is reported in accord with the Surgical Case Report (SCARE) 2018 Guidelines [9].

## 2 Case

A 25-year-old patient was referred to our multidisciplinary DSD team due to primary amenorrhea and ambiguous genitalia. The patient was raised as a female. Previously, she did not pay any attention to the absence of menses due to her parents saying that there were many women having similarly late first menstruation. However, she wanted to undergo examination due to her plan to be married that year.

**Fig. 1:**
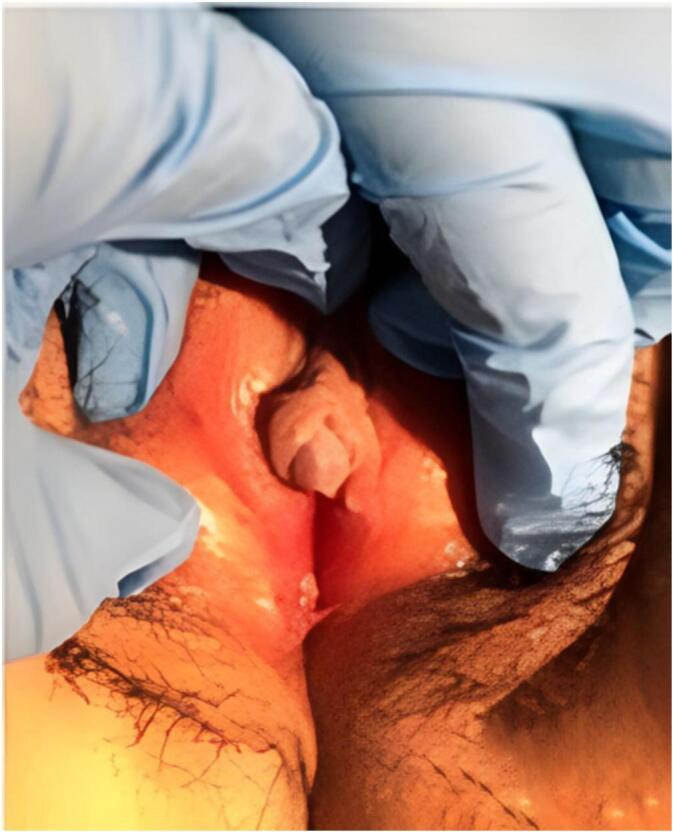
External genitalia appearance of the patient showing ambiguous genitalia.

The patient came with her older sister to our clinic. The patient's vital sign was stable at the time of arrival. Physical examination revealed that her tanner stage was in accordance with stage III. A phallic structure similar to clitoral hyperplasia with size of 1 × 1.5 cm was found, a feature suggesting an apparent female DSD. Her external urethral orifice was found right under her phallic structure. A probe was inserted and obstructed after 4 cm of insertion. There was no palpable gonad on both labia and no labioscrotal separation (Fig. 1).

Physical examination revealed no other pathology on bladder and kidney. She had no scrotal and testicular structure at her vulvar region. Complete blood count, hormonal level, karyotyping, and magnetic resonance imaging were done following the meeting. Her complete blood count was within normal limit. Her FSH and LH levels were increased, her estradiol level was reduced, while her testosterone level was normal, signifying a gonadal failure. However, her karyotyping examination revealed that she had 46, XY (male) karyotype.

**Fig. 2:**
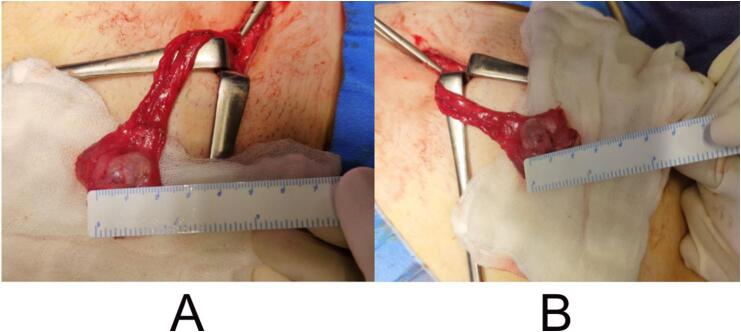
Bilateral orchiectomy results showing bilateral structures resembling testes (A) left gonad (B) right gonad.

Ultrasound examination revealed no testicular structures on her major labia, inguinal, and pelvic area. Furthermore, there was no visualization of the uterus. Based on the magnetic resonance imaging results, it was found that she did not have any uterus or vagina, while she had bilateral structures resembling testes at bilateral inguinal region (Fig. 2). Diagnostic laparoscopy was performed, and a gonadal structure was found on both inguinal area with size of three millimeters times 2 mm. Based on the examinations, she was diagnosed as having complete androgen insensitivity syndrome.

Psychiatric examination was also performed on the patient. It was found that there were no severe psychological disorders, but there were symptoms of adjustment disorders for the past 3 months due to her becoming aware of her diagnosis and tedious management of her condition. Fortunately, her condition did not create an obstacle for her daily functions.

Following the examinations, several family meetings was held involving the patient, her partner, her sister, her parents, the DSD team, and religion leader to discuss the results of her examinations and the most optimal management. The patient said that she identified herself as a woman and she was also raised as a woman. However, she was deeply bothered by the results of her examination, especially her male karyotype and the fact that she had testes while not having uterus as a “normal” female. Moreover, she had already had a partner (boyfriend) for 2 years and planned to be married to her partner later that year.

The patient said that during her puberty period, she was rather shy due to not having normal breast development and no menses. Therefore, she only had a few friends. She had never consulted her friend, both at her school and work, regarding her problems. She was also attracted to men, but she was unable to express her feeling due to her shyness and her social circle considering the act of women approaching men first as unusual.

Following the examinations, she had resigned from her work as she had to attend monthly follow-up examinations. Currently, she was confused about her gender identity and asked for both medical and psychological guidance for her condition. Moreover, as a Moslem, she was afraid that her prayers since childhood were not accepted as she was praying as a woman, but her examination results said otherwise. Finally, it was decided that she would remain as a woman, while her external genitalia would be altered to appear as a female. Both the psychiatric department and forensic department had been consulted and agreed to the decision.

**Fig. 3:**
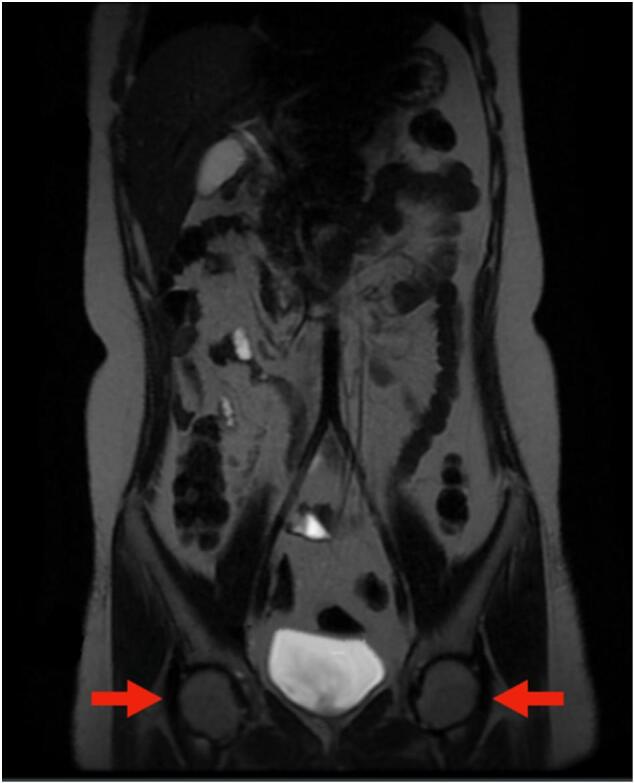
Magnetic resonance imaging result of the patient showing bilateral testes.

Laparoscopic bilateral orchiectomy and clitorovaginoplasty were planned after the family meetings. During the investigation, bilateral structures resembling testes were found at the inguinal region (Fig. 3). Histology examination results on the structures' tissue were in accordance to dysgenetic testes.

**Fig. 4:**
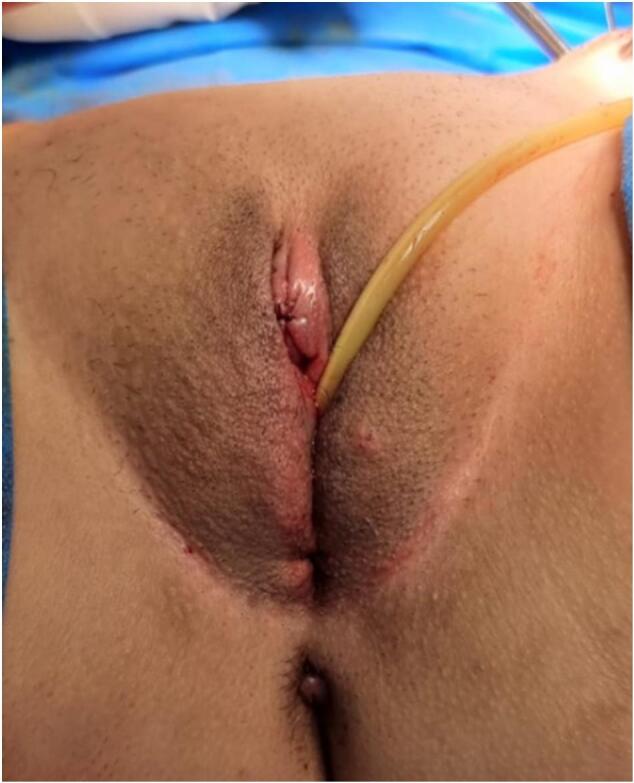
Final appearance of the external genitalia.

Following clitorovaginoplasty, a functional vagina with 7 cm of vaginal length was created. She was also prescribed with estradiol for hormonal therapy (Fig. 4). Follow-up examination one month after the surgeries revealed that the patient and her partner had not engaged in sexual activity. However, there were no complications or complaints from the patient. A year following the surgery, the patient had married her partner and already performed sexual activity with an intensity of once per two to three days. They had also achieved sexual satisfaction using the neovagina.

## 3 Discussion

Disorders of sexual development (DSD) are complicated problems requiring thorough examination and multidisciplinary approach [2]. As the term includes those appearing as male, female, and neither, each case of DSD is unique and requires tailored management. The case in our study is a complete androgen insensitivity (CAIS) case. CAIS usually becomes evident during adolescence when primary amenorrhea occurs despite normal breast growth. In some cases, CAIS can also manifest in infancy as detectable inguinal masses in a person with a 46,XY karyotype and an external female genital appearance. Due to the absence of target organ for testosterone produced by the gonads, the testosterone is fully converted to estradiol, such as in our case [10].

Androgen has an important role in both the reproductive and non-reproductive male functions throughout life. Androgen mainly depends on the direct interaction with the androgen receptor (AR), which is encoded by the AR gene contained on the long arm of the X chromosome. Mutation in AR may lead to deficit or alteration in AR synthesis, to inability to bind with the ligand [11]. Complete androgen insensitivity case is caused by inactivating mutation in the AR gene, creating a complete resistance to the action of the androgen, determining a female phenotype in persons with 46,XY karyotype and function testes [12]. Until now, the management of CAIS remains poorly standardized. Moreover, the condition is more aggravated in countries with limited infrastructure [12].

Along with biological problems such as hypospadias, undescended testes, clitoromegaly, and ambiguous genitalia, behavioral and psychological problems are common among those affected with DSD [4,8,13]. Those problems are especially obvious on patients who are diagnosed later in life, as they have established their gender identity prior to the examination [14,15]. Gender identity disorders (gender dysphoria) are also common among those affected with DSD patients as they are confused whether they are men or women [16]. Desire to belong to a gender, sometimes changing multiple times throughout life, is a widespread problem requiring serious attention from clinicians worldwide. In our case, the patient had only been diagnosed in her twenties, months before her marriage. This condition would surely put a tremendous pressure on her as both of her identity and her marriage were at risk.

Social norms and traditions also played a major part in determining the choices and outcomes available for patients with DSD. The first impact of the social norms and traditions effect to the CAIS patients is delayed diagnosis and therapy. Even in developed countries, the diagnosis of CAIS is often delayed until primary amenorrhea during puberty [12]. Moreover, the perception of “normal” marriage and fertility played a major concerns for DSD patients, as marriage is often seen as a precondition for becoming a fully respected member of the society [4]. The European Association of Urological (EAU) Guidelines on Paediatric Urology 2023 shows that a lower quality of life, especially in the domain of social relationship, is found in adult patients with DSD [2]. A previous study in Indonesia has shown that adult 78 % of adult women in Indonesia with DSD never have romantic/sexual relationships due to taboo, fear of ostracism, and expected infertility [17].

The age of which the diagnosis of DSD is made is a variable with conflicting effect on DSD patients. Previous study has shown that the diagnosis at the later art of life is associated with better psychological outcomes due to a more stabilized identity and personality constructed prior the diagnosis. However, other study also shows that late diagnosis is associated with increased psychological stress [18]. A significant increase of emotional and behavioral problems was also found in women with atypical genitalia, compared to DSD patients with conformity of sexual phenotype and assignment [18].

The tendency to seek social acceptance presents in every human. Contrary to some countries which have already recognized non-binary gender, most Indonesians only accept binary gender categories [19]. It is important to distinguish and separate male and female in the form of clothing, activities, and even religious activities in the social setting [4,17].

The diagnosis of DSD is usually considered not as a medical emergency, but a social emergency [3]. A previous study even stated that complete hormonal analysis was not performed on DSD patients originating from low-resource countries [20]. The lack of basic diagnostic and treatment for DSD patients is coupled with negative cultural representation of the condition, aggravating the condition for DSD patients [20]. Thus, social and religious support is important and beneficial for patients with DSD, especially through gender assignment process.

Another problem regarding patients with DSD is fertility. There are types of DSD with preserved fertility, leading to a hope that fertility is a possible outcome [4,21]. However, in complete androgen insensitivity syndrome such as in our case, there is an absence of Mullerian structures, rendering fertility impossible. Until now, there is no established or proven method of fertility preservation in complete androgen insensitivity syndrome case [10]. This information may create another stress factor for both of the patient, her partner, and her family [4]. It is especially important in a collective-driven society that expects procreation [4]. Therefore, psychological management from a psychologist or psychiatrist who is familiar with DSD cases is extremely valuable in managing DSD cases [22].

## 4 Conclusion

Disorders of sexual disorders are not only a biological problem, but also a psychological, social, and ethical problem. Comprehensive and multidisciplinary approach is essential for managing DSD patients.

## Informed consent

Written informed consent was obtained from the patient for anonymized patient information to be published in this article.

## Previous presentation

Authors declare that the study has never been presented on any scientific presentation before.

## Ethical approval

The ethics for this research project has been approved by a suitably constituted Ethics Committee of the institution and it conforms to the provisions of the Declaration of Helsinki. The ethical clearance was issued by the Research Ethics Committee of Faculty of Medicine, University of Indonesia, approval letter number KET-830/UN2.F1/ETIK/PPM.00.02/2022. The informed consent was obtained from the subject and the family members.

## Funding

The funding for this study was obtained via a research grant provided by 10.13039/501100006378Universitas Indonesia.

## Guarantor

Kevin Yonathan.

## Research registration number

Not applicable.

## CRediT authorship contribution statement

**Irfan Wahyudi:** Conceptualization, Acquisition of data, Formal analysis, Investigation, Resources, Writing, Supervision, Funding acquisition. **Kevin Yonathan:** Methodology, Software, Writing, Validation, Project administration. **Arry Rodjani:** Conceptualization, Investigation, Resources, Writing, Supervision, Funding acquisition. **Gerhard Reinaldi Situmorang:** Resources, Writing, Supervision.

## Declaration of competing interest

The funding for this study was obtained via a research grant provided by 10.13039/501100006378Universitas Indonesia. The authors declare no other conflict of interests in this study.
